# Vulnerability to the transmission of human visceral leishmaniasis in a Brazilian urban area

**DOI:** 10.1590/S1518-8787.2017051006532

**Published:** 2017-05-08

**Authors:** Celina Roma Sánchez de Toledo, Andréa Sobral de Almeida, Sergio Augusto de Miranda Chaves, Paulo Chagastelles Sabroza, Luciano Medeiros Toledo, Jefferson Pereira Caldas

**Affiliations:** I Divisão de Vigilância em Saúde. Secretaria Municipal de Saúde do Rio de Janeiro. Rio de Janeiro, RJ, Brasil; II Departamento de Endemias Samuel Pessoa. Escola Nacional de Saúde Pública. Fundação Oswaldo Cruz. Rio de Janeiro, RJ, Brasil; III Programa de Pós-Graduação em Epidemiologia em Saúde Pública. Escola Nacional de Saúde Pública. Fundação Oswaldo Cruz. Rio de Janeiro, RJ, Brasil

**Keywords:** Leishmaniasis, Visceral, epidemiology, Health Vulnerability, Socioeconomic Factors, Ecological Studies, Leishmaniose Visceral, epidemiologia, Vulnerabilidade em Saúde, Fatores Socioeconômicos, Estudos Ecológicos

## Abstract

**OBJECTIVE:**

To analyze the determinants for the occurrence of human visceral leishmaniasis linked to the conditions of vulnerability.

**METHODS:**

This is an ecological study, whose spatial analysis unit was the Territorial Analysis Unit in Araguaína, State of Tocantins, Brazil, from 2007 to 2012. We have carried out an analysis of the sociodemographic and urban infrastructure situation of the municipality. Normalized primary indicators were calculated and used to construct the indicators of vulnerability of the social structure, household structure, and urban infrastructure. From them, we have composed a vulnerability index. Kernel density estimation was used to evaluate the density of cases of human visceral leishmaniasis, based on the coordinates of the cases. Bivariate global Moran’s I was used to verify the existence of spatial autocorrelation between the incidence of human visceral leishmaniasis and the indicators and index of vulnerability. Bivariate local Moran’s I was used to identify spatial clusters.

**RESULTS:**

We have observed a pattern of centrifugal spread of human visceral leishmaniasis in the municipality, where outbreaks of the disease have progressively reached central and peri-urban areas. There has been no correlation between higher incidences of human visceral leishmaniasis and worse living conditions. Statistically significant clusters have been observed between the incidences of human visceral leishmaniasis in both periods analyzed (2007 to 2009 and 2010 to 2012) and the indicators and index of vulnerability.

**CONCLUSIONS:**

The environment in circumscribed areas helps as protection factor or increases the local vulnerability to the occurrence of human visceral leishmaniasis. The use of methodology that analyzes the conditions of life of the population and the spatial distribution of human visceral leishmaniasis is essential to identify the most vulnerable areas to the spread/maintenance of the disease.

## RESUMO

**OBJETIVO:**

Analisar determinantes para a ocorrência da leishmaniose visceral humana vinculados às condições de vulnerabilidade.

**MÉTODOS:**

Estudo ecológico, cuja unidade de análise espacial foi a Unidade de Análise Territorial em Araguaína, TO, de 2007 a 2012. Foi realizada análise da situação sociodemográfica e de infraestrutura urbana no município. Indicadores primários normalizados foram calculados e utilizados na construção de indicadores de vulnerabilidade de estrutura social, de estrutura domiciliar e de infraestrutura urbana. A partir deles, foi composto um índice de vulnerabilidade. A estimativa de *Kernel* foi utilizada para avaliar a densidade de casos de leishmaniose visceral humana, com base nas coordenadas dos casos. O *I-Moran* Global Bivariado foi empregado para verificar a existência de autocorrelação espacial entre a incidência de leishmaniose visceral humana e os indicadores e índice de vulnerabilidade. *I-Moran* Local Bivariado foi utilizado para identificar *clusters* espaciais.

**RESULTADOS:**

Foi observado um padrão de disseminação centrífuga da leishmaniose visceral humana no município, em que surtos da doença atingiram progressivamente áreas centrais e periurbanas. Houve correlação entre maiores incidências de leishmaniose visceral humana e piores condições de vida. Foram observados *clusters* estatisticamente significativos entre as incidências de leishmaniose visceral humana nos dois períodos analisados (2007 a 2009 e 2010 a 2012) e os indicadores e índice de vulnerabilidade.

**CONCLUSÕES:**

O ambiente em áreas circunscritas contribui como fator de proteção ou aumenta a vulnerabilidade local à ocorrência de leishmaniose visceral humana. O uso de metodologia que analisa as condições de vida da população e distribuição espacial da leishmaniose visceral humana é essencial na identificação de áreas mais vulneráveis à disseminação/manutenção da doença.

## INTRODUCTION

Visceral leishmaniasis (VL) is a vector-borne parasitic disease whose importance in the context of public health in Brazil has increased significantly in recent years. This fact is mainly due to the expansion of urbanization processes and the modification of habitats of the species involved in the transmission cycle^5,22^.

The geographical areas with transmission of VL are increasingly expanding. The disease, considered one of the priorities among tropical diseases by the World Health Organization (WHO), is endemic in ninety-eight countries, including Brazil^a^, whose areas of occurrence increase progressively^9,13^. There is a great gap in scientific knowledge about VL. Studies are needed on new drugs and therapeutic regimes, as well as studies that address the effectiveness of disease control actions with greater methodological strength, among others aspects^22^.

Araguaína, in the State of Tocantins, Brazil, presents a recent endemic-epidemic process of VL. There has been an alarming increase in the number of confirmed human cases mainly between 2006 (56) and 2007 (251), when it presented the greatest number of records of the disease in Brazil (7.0% of all reported cases in the country). Since then, it remains among the four Brazilian municipalities with the highest absolute number of annual records of VL.

The analysis of the vulnerability^20^ of the region for the transmission of VL using indicators and the analysis of the spatial distribution of urban VL can contribute to the better understanding of the heterogeneous transmission process, resulting from the socio-territorial organization of the municipality^1,2,5^.

This study aimed to analyze the determining circumstances for the occurrence of human visceral leishmaniasis (HVL), linked to the conditions of vulnerability in Araguaína. The data can provide elements for the formulation of public policies adapted to this situation.

## METHODS

**Figure 1:**
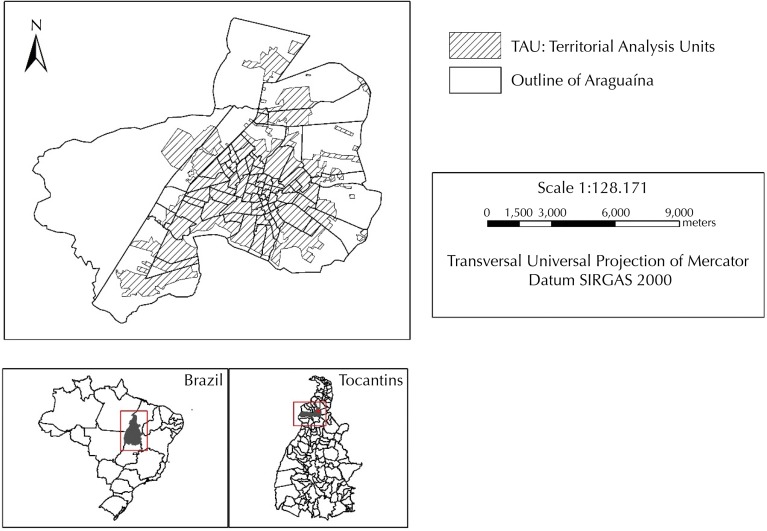
Maps with the Territorial Analysis Units (TAU). Araguaína, State of Tocantins, Brazil, 2010.

Araguaína (Figure 1) is located in northern Brazil, in the northern portion of the State of Tocantins. It has an altitude of 227 m, under the geographical coordinates 7°11’28” South latitude and 48°12’26” West longitude. It is 380 km from Palmas, capital of the State (IBGE, 2014). It presents high temperatures throughout the year, approximately 28°C. The population distribution is predominantly urban, with great rural emptiness. Between the 2000 and 2010 Demographic Censuses (Brazilian Institute of Geography and Statistics – IBGE)^b,c^, we can observe a growth of 33% (from 113,143 to 150,484 inhabitants) of the total population of the municipality and approximately 35% of the urban population (from 105,874 to 142,925 inhabitants).

The urban territory of Araguaína is characterized by a central region with high density and intermediate and peripheral areas, discontinuous among themselves. The growth model of this municipality is discontinuous and segregated. For the most part, the areas with increased population density have vast unoccupied areas with considerable spacing between the buildings. In addition, most of the streets in these areas are not paved.

This is an ecological study, with cases of HVL notified between 2007 and 2012 in Araguaína residents. The cases reported and confirmed in residents were obtained from the Municipal Health Department of the Municipality and the *Sistema de Agravos de Notificação* (SINAN – System of Reportable Diseases)/ Department of Health Surveillance (SVS)/ Ministry of Health. The definition of confirmed case of VL is based on criteria adopted by the Ministry of Health to guide the actions of epidemiological surveillance of the disease throughout the country^15^.

We performed the analysis of the situation of vulnerability to the transmission of the disease based on the construction of indicators related to the conditions of urban, social, and household infrastructure for 2010, the analysis of the distribution of HVL in time and space in two three-year periods (2007-2009 and 2010-2012), in addition to the descriptive analysis of the cases of canine visceral leishmaniasis (CVL) notified by the Municipal Health Department between 2006 and 2008. The analysis in three-year periods was used because the disease has a cyclical nature. In addition, this approach helps to reduce the variability in the number of cases reported between the years of the study.

The digital base of the territorial analysis units (TAU) was made from the compatibility of the bases of urban census tracts of the 2000 and 2010 Demographic Censuses of the IBGE^d^. The compatibility was carried out because those geographical bases are very distinct (Figure 1).

We generated polygons of the areas occupied by the human population, based on the methodology referred to in the Land Use Manual^11^. The polygons of the occupied areas were vectorized by the method of visual interpretation of satellite images. We identified the spaces with urban characteristics at a 1:5,000 scale, using geoprocessing tools in a Georeferenced Information System (GIS). This step was developed based on the Digital Globe satellite images for 2014, obtained by the software Google Earth, with spatial resolution of one meter. The software used was ArcGis 10.2.

The autochthonous cases of HVL reported between 2007 and 2012 were georeferenced from the address found in the database of the SINAN, with the use of Google Earth. This generated a digital data mesh.

The mesh containing the specific data relating to the cases of the disease was superimposed to the digital base of TAU and the areas occupied by human populations in each TAU. This allowed the aggregation of cases of HVL based on these units of territorial analysis.

For the calculation of incidence of the two three-year periods (2007-2009 and 2010-2012), we used the cases reported and confirmed in the periods in the numerator and the population obtained from the population projection per TAU, based on the 2000 and 2010 Census populations, in the denominator. The calculation of the density of cases was carried out based on the area actually occupied by the human population of the TAU.

Kernel density estimation was used to evaluate the density of cases of HVL, based on the coordinates of the cases. Thematic maps were prepared for the spatial and temporal analysis of the evolution of the endemic disease, as well as their correlation with the information about the situation of social and household structure and urban infrastructure. The application ArcGis 10.2 was used for this analysis.

Official information, available on the website of IBGE on the 2010 Demographic Census, was used for the analysis of the conditions of urban, social, and household infrastructure of the municipality. This analysis identified the most vulnerable areas in the urban and social infrastructure, in order to reveal differences between the various areas of the municipality.

Primary indicators were calculated based on the variables of the 2010 Demographic Census. Such indicators had their values normalized according to the methodology used by Tiburcio and Corrêa^20^. We applied T1 (positive indicators: high values represent lower vulnerability situations) and T2 (negative indicators: low values represent lower vulnerability situations) equations. The highest values observed (*Maximum*I), the lowest values observed (*Minimum*_I_), and the individual value of each primary indicator (*I*_Observed_) were used for the calculation:


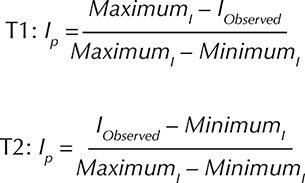


Indicators of vulnerability of social structure (IVSS), household structure (IVHS), and urban infrastructure (IVUI) were created from normalized indicators. The indicators had their values converted into scores that ranged from 0 to 1, with cutoff points defined according to quartiles of distribution, assigning the lowest values to the best conditions (Table 1). The composite vulnerability index (CVI) was prepared based on these indicators of vulnerability. The CVI was prepared by the arithmetic average of the three indicators of vulnerability (IVSS, IVHS, and IVUI).

**Table 1:** Primary indicators used in the construction of the indicators of vulnerability and composite vulnerability index. Araguaína, State of Tocantins, Brazil.

Vulnerability index	Vulnerability indicators	Primary indicators
CVI	IVSS	Density per room (T2) Proportion of literate persons responsible for the household (T1) Proportion of literate persons aged eight years or more (T1) Dependency ratio (T2)
	IVHS	Proportion of PPH with water supply (T1) Proportion of PPH with bathroom of exclusive use of the residents or health and sanitation from the sewer network (T1) Proportion of PPH with garbage collected by the municipality (T1) Proportion of PPH with electricity from the utility company (T1)
	IVUI	Proportion of PPH with street lighting in the surroundings (T1) Proportion of PPH with paved streets in the surroundings (T1) Proportion of PPH with sewer/manhole in the surroundings (T1) Proportion of PPH with trees in the surroundings (T1)

CVI: Composite vulnerability index; IVSS: Indicator of vulnerability of social structure; IVHS: Indicator of vulnerability of household structure; IVUI: Indicator of vulnerability of urban infrastructure; PPH: Permanent private households; T1: (positive indicators) high values represent lower vulnerability situations; T2: (negative indicators) low values represent lower vulnerability situations

The IVSS was created to combine the information from various sociodemographic indicators into a synthetic indicator to establish an order in relation to the level of social vulnerability. The IVHS was prepared with the information of various indicators of household structure, allowing a synthetic measure of the housing conditions of the population. The IVUI was created to synthesize the information on the urban infrastructure surrounding the household into a single indicator.

Bivariate Global Moran’s I was used to verify the existence of spatial autocorrelation between the incidence rate of HVL and the Vulnerability Indices. The index tests whether the values of an indicator in a given region is related to the values of another variable in neighboring regions. Statistics can have the values from -1 to +1, positive for direct correlation and negative when inverse^3,16,e^. Bivariate Global Moran’s I does not show where the spatial cluster are. Thus, we used Bivariate Local Moran’s I, which provides the degree of spatial autocorrelation, statistically significant, in each regional unit. According to Anselin, this index provides an indication of the degree of linear association between the value for a variable in a given area and the average of another variable in the surrounding areas. The matrix of the neighborhood W defined by contiguity was the Queen matrix, which considers two neighboring regions that have common borders, in addition to common nodes (vertices).

The statistical significance of Moran’s I Index was tested using permutations. We generated subsequent permutations of the values of the data associated with the TAU. Each permutation produces a new spatial arrangement, in which values are distributed randomly between the TAU. That way, we can create an empirical distribution of statistics I under the null hypothesis of spatial randomness. The empirical pseudo-significance was based on ninety-nine random permutations. The program used in this analysis was GeoDa 1.6.6^3^.

The information about the cases of CVL was provided according to neighborhood by the Center of Control of Zoonosis (CCZ) of Araguaína. As there was incompatibility of the sketch of neighborhoods with the digital cartographic bases of the census tracts and TAU, the analyses were restricted to the municipal level. We calculated the positivity of cases of CVL from the absolute number of samples collected and the number of samples per year.

This study has been approved by the Research Ethics Committee of the *Escola Nacional de Saúde Pública Sérgio Arouca* of the *Fundação Oswaldo Cruz* (Protocol 142/11).

## RESULTS

We observed an increase of approximately 30% of the resident population in the urban area of the municipality of Araguaína between the 2000 and 2010 Censuses. We could also identify a more marked growth (6.7%) in the population aged 60 years or more and a less accentuated growth (5.9%) among individuals aged between zero and four years. The illiteracy rate of the population aged 15 years or more decreased in the same period (13.4%, 2000 Census; 8.6%, 2010 Census), as well as the proportion of permanent private homes with sanitation considered by the IBGE as inadequate (7.5%, 2000 Census; 4.7%, 2010 Census).

We found a significant number of cases of the disease in the municipality during the period, although there was a decrease of 44.6% when we compared the first year (2007) of the historical series analyzed with the last one (2012). This decrease followed what was observed in the country to a lesser degree (decrease of 4.8% between 2007 and 2012). Approximately 251 cases were reported in 2007, 262 cases in 2008, 164 cases in 2009, 96 cases in 2010, 184 cases in 2011, and 139 cases in 2012. Two epidemic waves were observed in the city; the first with a peak in 2008 and the second one with a peak in 2011. Epidemic waves were defined based on the significant increase in the number of human cases of VL when observed the historical series.

Of the 1,096 cases of HVL notified and confirmed between 2007 and 2012, 84.8% were georeferenced. Georeferencing losses occurred because of the incompleteness of the address field in the SINAN or because we could not properly locate the address on Google Earth. The confirmed cases of VL were superimposed to the digital cartographic base of the occupied areas in the TAU to observe the dispersion of the disease throughout the municipality.

**Figure 2:**
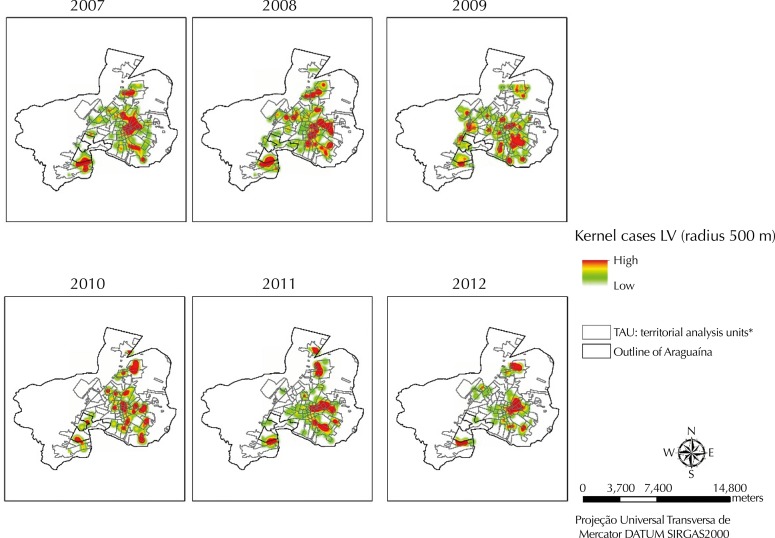
Kernel Map of cases of visceral leishmaniasis (VL) in the occupied areas of the territorial analysis units I. Araguaína, State of Tocantins, Brazil, 2007 to 2012.

The areas with greater intensity of occurrence of HVL in Araguaína were estimated using the Kernel method (Figure 2). We observed a more expressive cluster in the central urban area and other smaller clusters in the peripheral areas of the municipality in the first year of the period. The centrifugal spread of the disease, with greater spreading of cases throughout the territory, occurred over time. A pattern close to the one for 2007 was observed in the last two years of the period.

* Areas occupied by human population.

**Figure 3:**
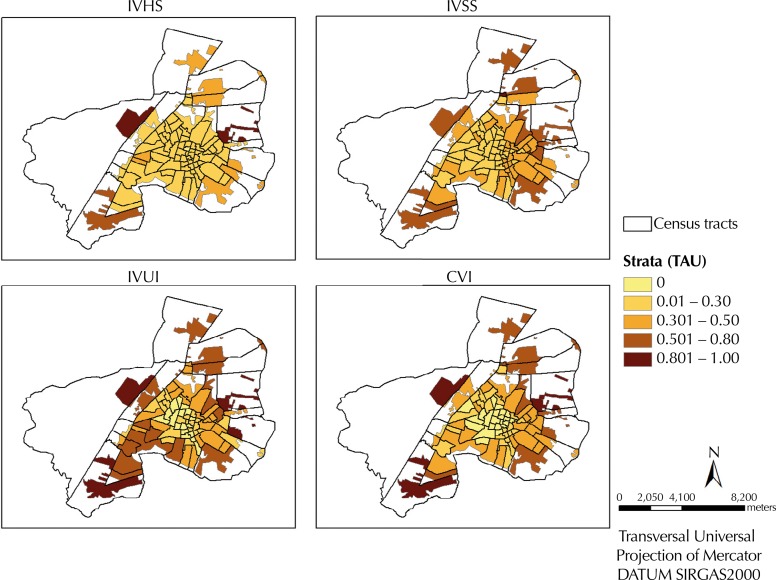
Indicators of specific vulnerability (IVHS, IVSS, IVUI) and composite index (CVI) according to the scores of the distribution in occupied areas. Araguaína, State of Tocantins, Brazil, 2007 to 2012.

IVHS: Indicator of vulnerability of household structure; IVSS: Indicator of vulnerability of social structure; IVUI: Indicator of vulnerability of urban infrastructure; CVI: Composite vulnerability index; TAU: Territorial analysis units

The worst scores of IVSS, IVHS, and IVUI are located in the occupied areas of the outskirts of the municipality (Figure 3). The IVUI had the worst score values (close to one), showing the precarious conditions of the urban infrastructure in most of the area analyzed.

The indicators of vulnerability with greater significant global spatial autocorrelation (Bivariate Moran’s I; p value) with the incidence of HVL of the two periods, respectively, were: IVSS (I = 0.132554; p = 0.05 – I = 0.221152; p = 0.03) and IVUI (I = 0.127157; p = 0.03 – I = 0.148091; p = 0.04). The IVHS had spatial autocorrelation only for the incidence of HVL of the first period, while CVI had spatial autocorrelation for both periods (I = 0.140858; p = 0.04 – I = 0.143946; p = 0.01) (Table 2).

**Table 2:** Index of correlation of Bivariate Moran’s I between the incidence of visceral leishmaniasis in the periods of 2007–2009 and 2010–2012 and indicators and index of vulnerability. Araguaína, State of Tocantins, Brazil, 2010.

Incidence rate	IVHS		IVSS		IVUI		CVI	
			
	Biv Moran’s I	p	Biv Moran’s I	p	Biv Moran’s I	p	Biv Moran’s I	p
Inc 07_09	0.093865	0.05	0.132554	0.05	0.127157	0.03	0.140858	0.04
Inc 10_12	-0.02625	0.41	0.221152	0.03	0.148091	0.04	0.143946	0.01

IVSS: Indicator of vulnerability of social structure; IVHS: Indicator of vulnerability of household structure; IVUI: Indicator of vulnerability of urban infrastructure; CVI: Composite vulnerability index

**Figure 4:**
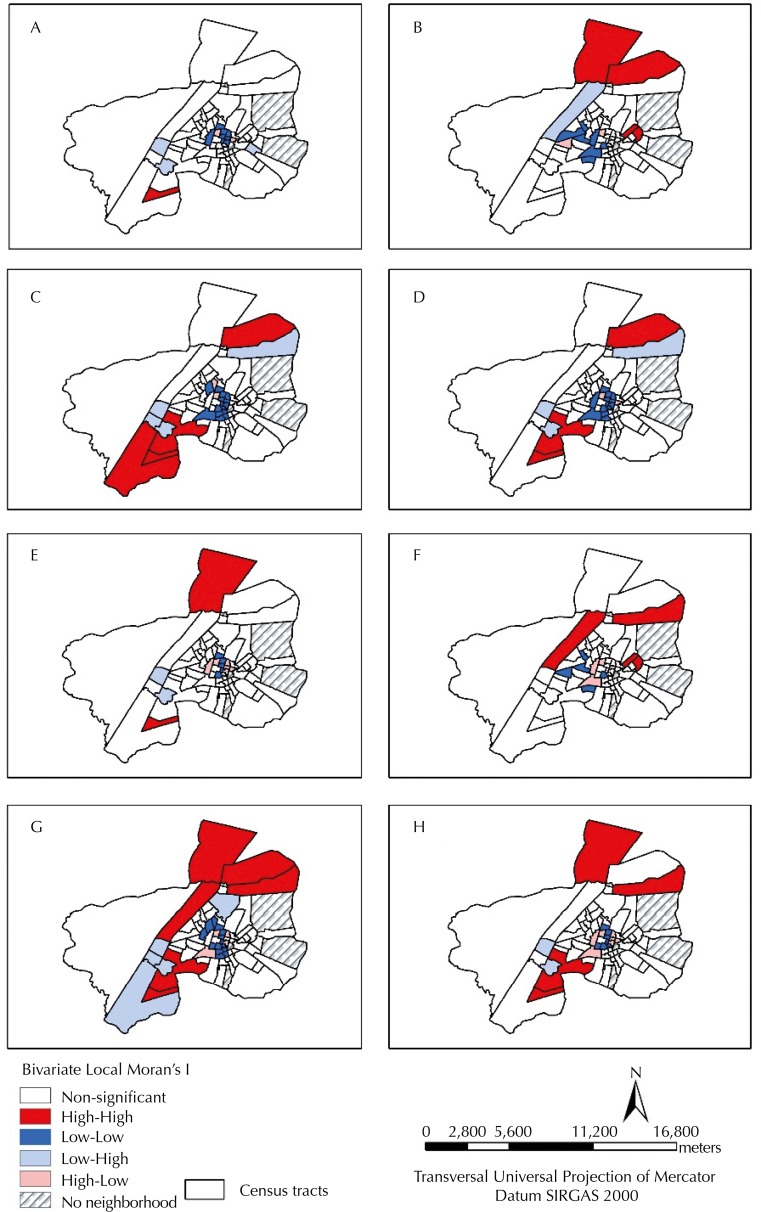
Maps of the index of correlation of Bivariate Local Moran’s I between the incidence of visceral leishmaniasis and indicators of specific vulnerability (A, B, C, D, E, F, G) and composite vulnerability index (D, H). Araguaína, State of Tocantins, Brazil, 2010.

Bivariate Local Moran’s I showed the presence of statistically significant clusters between the incidences of HVL in both periods and the indicators of vulnerability for the census tracts (Figure 4).

A: Incidence (2007-2009) *versus* Indicator of vulnerability of household structure (IVHS).

B: Incidence (2007-2009) *versus* Indicator of vulnerability of social structure (IVSS).

C: Incidence (2007-2009) *versus* Indicator of vulnerability of urban infrastructure (IVUI).

D: Incidence (2007-2009) *versus* composite vulnerability index (CVI).

E: Incidence (2010-2012) *versus* IVHS.

F: Incidence (2010-2012) *versus* IVSS.

G: Incidence (2010-2012) *versus* IVUI.

H: Incidence (2010-2012) *versus* CVI.

Main clusters (Low-Low in central areas and High-High in the northern and southern peripheral regions) were evidenced, as well as intermediate clusters (Low-High and High-Low) in all combinations tested in the period analyzed (Figure 4). The main clusters located in the central region showed areas of low incidence of HVL circumscribed by better living conditions (those with low scores), while the peripheral clusters revealed areas of high incidence of HVL circumscribed by areas with worse living conditions (those with high scores). The High-High clusters located in the southern peripheral region of the municipality remained virtually unchanged in both periods for the indicators IVUI and CVI (Figure 4).

No significant variations were observed regarding canine seropositivity: 33% was observed in 2006, 37.9% in 2007, and 37.1% in 2008.

## DISCUSSION

We observed a pattern of centrifugal spread of human visceral leishmaniasis in Araguaína. Outbreaks of the disease reached progressively, from 2008, the occupied central and peri-urban areas of the municipality. However, the pattern of spread had important annual intensity variations.

Other Brazilian municipalities, also in the process of increasing urbanization and precarious living conditions, have different epidemiological patterns of spread of VL. Among these patterns, we can identify: restricted peri-urban pattern (outbreaks of the disease restricted to areas of the urban periphery), diffuse peri-urban pattern (outbreaks of the disease mainly affecting the urban peripheries and, secondarily, central/urbanized areas), epidemiological pattern of centrifugal spread (outbreaks of the disease spread within the municipality, and also, increasingly, in neighboring municipalities), and, finally, pattern of network dissemination (outbreaks of the disease sequentially affecting interconnected municipalities)^1,4,6,13,18^. Few municipalities have a rural epidemiological pattern, dominated by rural outbreaks with occurrence of sporadic human cases^19^.

The centrifugal process of spread of the disease in Araguaína is characterized by important changes in the territory, such as: disorderly expansion of the city – which has advanced through the natural habitat of the vector of VL –, lack of basic and sanitary infrastructure – which enable the urbanization of the vector –, and the spread of the disease in the municipality^5,7,17,18,21^.

The increase of approximately 30% of the population living in the municipality between the 2000 and 2010 Censuses and the changes of the urban space that are inherent to it can help to explain the maintenance of the high number of human cases of VL notified in the historical series (2007-2012), although we can observe a wide variation in the number of reported cases over the years. There was an increase of susceptible and vulnerable persons inserted in that territory, as well as individuals – migrants and their animals, for example – from endemic areas for VL^8,14,f^.

Although the notification of cases of VL is the main strategy for surveillance of the disease in Brazil, the process of notification and confirmation of cases can be slow. The HVL is considered a mandatory notifiable disease in the country since 1975. Its vigilance is decentralized and especially uses the data of notifications recorded in the SINAN. The analysis of these data allows the space-temporal monitoring of the disease in the country, subsidizing the actions for its control^15^.

A correlation was observed between higher incidences of HVL and worse living conditions, although it was not high. Although the TAU presented certain uniformity regarding the indicators analyzed, we could observe clusters from the calculation of Bivariate Local Moran’s I. This approach identified that the environment in circumscribed areas contributes as a factor of protection or increase for the relative local vulnerability concerning HVL in specific locations in Araguaína^17^. This reinforces the theory that the occurrence of HVL is closely related to the situation of social vulnerability^g^ in which parts of the population are inserted^18,g^.

The use of indicators of vulnerability regarding the territory contributed to the knowledge on the intra-urban differences more associated with the occurrence of HVL. The analysis of spatial variability based on the combination of these indicators of vulnerability and the incidence of VL allowed the identification of particularly distinct areas, i.e. with their own socioeconomic characteristics that can contribute to the process of reproduction of the disease in the municipality. Such areas should be the target of local public policies aiming to reduce the vulnerability and decrease the number of cases of the disease in the territory of Araguaína^20,h^.

The occupation of the urban space arises from the actions of society on the nature. Its configuration incorporates social and economic structure and its dynamics, establishing the flows of the local movement of goods and services.

The lack of consistent data about the canine reservoir is one of the limitations of the study. We highlight the annual variation of the areas included in the canine serological surveys conducted by the CCZ of Araguaína and the lack of regularity of the survey in each area for the years of study. The variation of the number of dogs included in the surveys annually has not allowed further evaluations on the seroprevalence of VL.

The scenario of visceral leishmaniasis in Brazil is complex. Researchers need to seek new methodologies of analysis that can cover the various elements involved in the process of introduction, dissemination, and maintenance of the disease. The difficulties related to its control must be considered, from the control of the canine population and vectors and the identification of infected animals up to the elimination of risk factors.

The use of methodologies that enable the analysis of the living conditions of the population, along with the spatial distribution of human visceral leishmaniasis, becomes essential to identify the areas that present greater vulnerability for the spread and maintenance of the disease in the territory^2,10,17^.

We believe that this approach may contribute to the definition of control strategies for VL. It can reduce not only operating costs but also provide increased effectiveness of the actions of surveillance and control of this endemic disease.

## Footnotes

a World Health Organization. Global Health Observatory (GHO) Data. Leishmaniasis: situation and trends. Geneva: World Health Organization; 2010 [cited 2010 Oct 28]. Available from: http://www.who.int/gho/neglected_diseases/leishmaniasis/en/

b Instituto Brasileiro de Geografia e Estatística. Censo demográfico 2000: características da população e dos domicílios: resultados do universo. Rio de Janeiro: IBGE; s.d. [cited 2017 Feb 21]. Available from: http://www.ibge.gov.br/home/estatistica/populacao/censo2000/defaulttab_brasil.shtm

c Instituto Brasileiro de Geografia e Estatística. Censo demográfico 2010: resultados do universo. Rio de Janeiro: IBGE; s.d.[cited 2017 Feb 21]. Available from: http://www.ibge.gov.br/home/estatistica/populacao/censo2010/default_resultados_universo.shtm

d Instituto Brasileiro de Geografia e Estatística. Downloads: Geociências. Rio de Janeiro: IBGE; 2016 [cited 2017 Feb 21]. Available from: http://downloads.ibge.gov.br/downloads_geociencias.htm

e Anselin L. An introduction to spatial autocorrelation analysis with GeoDa. Urbana-Champaign: Univesity of Illinois; 2003 [cited 2014 Oct]. Available from: http://www.utdallas.edu/~briggs/poec6382/geoda_spauto.pdf

f Glória MRB. Leishmaniose visceral: situação epidemiológica e distribuição espacial, município de Palmas, Tocantins [dissertation]. Rio de Janeiro (RJ): Escola Nacional de Saúde Pública Sergio Arouca; 2006.

g Miranda GMD. Leishmaniose visceral em Pernambuco: a influência da urbanização e da desigualdade [dissertation]. Recife (PE): Centro de Pesquisas Aggeu Magalhães, Fundação Oswaldo Cruz; 2008.

h Araújo VEM. Análise da distribuição espaço-temporal da leishmaniose visceral e perfil clínico-epidemiológico dos casos e óbitos Belo Horizonte, Minas Gerais, 1994 a 2009 [these]. Belo Horizonte (MG): Universidade Federal de Minas Gerais; 2011.
